# Apathy in Alzheimer's Disease: Any Effective Treatment?

**DOI:** 10.1155/2014/421385

**Published:** 2014-02-02

**Authors:** Raffaele Rea, Anna Carotenuto, Angiola M. Fasanaro, Enea Traini, Francesco Amenta

**Affiliations:** ^1^Centro di Ricerche Cliniche, Telemedicina e Telefarmacia, Università di Camerino, 62032 Camerino, Italy; ^2^Unità Complessa Neurologia, Azienda Ospedaliera di Rilievo Nazionale A. Cardarelli, 80131 Naples, Italy

## Abstract

*Objective*. This review has evaluated the effectiveness of pharmacological treatment of apathy in patients with Alzheimer's disease (AD). *Methods*. A systematic literature search was conducted on published clinical trials assessing the effects of pharmacological treatment on apathy in AD over the last 10 years. *Results*. Fourteen studies considered of good quality were included in the analysis (4 randomized controlled trials, 9 open-label studies, and 1 retrospective analysis). Cholinesterase inhibitors were investigated in 9 studies, monoaminergic compounds such as methylphenidate and modafinil in two trials and one trial, respectively, and * Ginkgo biloba* (EGb 761 extract) and citalopram in one study each. Cholinesterase inhibitors did not show statistical significant effect in 1 RCT study but were associated to improvement in 3 open-label studies. Methylphenidate elicited a small but significant activity accompanied by relevant side effects such as high blood pressure, cough, and osteoarticular pain. EGb 761 was well tolerated and countered apathy. Other treatments induced modest improvements or were ineffective. *Conclusions*. Apathy treatment remains a challenge and there is no evident advantage of any specific pharmacotherapy tested so far. The development of controlled studies according to updated guidelines for the diagnosis of apathy in patients with AD is desirable.

## 1. Introduction

Apathy is a neurocognitive disorder characterized by the reduction of goal-directed behaviour. It may be found in different diseases and is common in neurodegenerative disorders. In Alzheimer's disease (AD) it has been found to be associated with specific atrophy patterns [1]. The first definition of apathy came in 1990 by Marins [2], who classified it as a motivational loss not attributable to emotional stress, cognitive impairment, or consciousness reduction [3]. Apathy has been subsequently related to disconnection of different brain circuitries, including the amygdala, which is innervated by cholinergic projections from the basal forebrain [4], thalamic nuclei of the midline, the basal forebrain, and the cholinergic pedunculopontine projections. The predominant cholinergic nature of nuclei probably disconnected in apathy suggests that impaired brain cholinergic neurotransmission is involved in apathy pathophysiology.

Recently apathy was classified from a clinical point of view into three different subtypes, namely, emotional affective apathy, cognitive apathy, and autoactivation apathy. Each of these forms of apathy is related to the impairment of specific processes [5]. Apathy due to the disruption of the *“emotional-affective”* processing is attributed to damage of circuits linking emotional-affective signals to ongoing or forthcoming behaviors. Lesions of the orbitomedial prefrontal cortex and/or related regions (limbic territory) of the basal ganglia (ventral striatum, ventral pallidum) are probably linked to this form of apathy. Apathy due to the disruption of the *“cognitive processing”* is attributed to impairment of circuits linking the planning of actions to the ongoing or forthcoming behaviors. Lesions of the dorsolateral prefrontal cortex and/or related regions (associative territory of the basal ganglia including the dorsal caudate nucleus) might be responsible for it. Apathy due to the disruption of *“autoactivation”* processing is attributed to damage of the circuits linking the self-activation of thoughts and actions to ongoing or forthcoming behaviors. Externally driven behaviors are relatively unaffected in autoactivation apathy. Lesions affecting bilaterally associative and limbic territories of the internal portion of the globus pallidus might be responsible for it [5]. From an epidemiological point of view apathy can be considered as a common symptom of AD. The frequency of it ranges from 19 to 76% [6], depending on the disease duration and stage and the age of subjects [7, 8].

The European Alzheimer's Disease Consortium has issued in 2008 guidelines for apathy diagnosis [9]. According to these guidelines for a correct diagnosis of apathy the diminished motivation clinical picture must persist for no less than four weeks, and two of the following three dimensions should be present: (i) reduced goal-directed behavior, (ii) reduced goal-directed cognitive activity, and (iii) reduced emotions. Moreover, functional impairment should be attributable to apathy [10]. Apathy is the cause of high distress levels in caregivers [11, 12], as they need to take continuous responsibility for everything. Over time, anger and conflicts inevitably follow between patients and caregivers. This makes apathy a risk factor for institutionalization.

*Diagnostic Criteria for Apathy Revised by European Alzheimer's Disease Consortium (EADC).* For a diagnosis of apathy the patient should fulfill criteria A, B, C, and D.

*Criterion A.* Loss of or diminished motivation in comparison to the patient's previous level of functioning is not consistent with his age or culture. These changes in motivation may be reported by the patient himself or by the observations of others.

*Criterion B.* It signifies the presence of at least one symptom in at least 2 of the 3 following domains for a period of at least 4 weeks and present most of the time.

*B1. Domain Behaviour.* Loss of or diminished goal-directed behaviour is evidenced by at least one of the following:

- initiation symptom: loss of self-initiated behaviour (e.g., starting conversation, doing basic tasks of day-to-day living, seeking social activities, and communicating choices);
- responsiveness symptom: loss of environment-stimulated behaviour (e.g., responding to conversation and participating in social activities).

*B2. Domain Cognition.* Loss of or diminished goal-directed cognitive activity is evidenced by at least one of the following:

- initiation symptom: loss of spontaneous ideas and curiosity for routine and new events (i.e., challenging tasks, recent news, social opportunities, and personal/family and social affairs);
- responsiveness symptom: loss of environment-stimulated ideas and curiosity for routine and new events (i.e., in the person's residence, neighborhood, or community).

*B3. Domain Emotion.* Loss of or diminished emotion is evidenced by at least one of the following:

- initiation symptom: loss of spontaneous emotion, observed or self-reported (e.g., subjective feeling of weak or absent emotions, or observation by others of a blunted affect);
- responsiveness symptom: loss of emotional responsiveness to positive or negative stimuli or events (e.g., observer reports of unchanging affect or of little emotional reaction to exciting events, personal loss, serious illness, and emotional-laden news).

*Criterion C.* These symptoms (A and B) cause clinically significant impairment in personal, social, occupational, or other important areas of functioning.

*Criterion D.* The symptoms (A and B) are not exclusively explained or due to physical disabilities (e.g., blindness and loss of hearing), to motor disabilities, to diminished level of consciousness, or to the direct physiological effects of a substance (e.g., drug of abuse and a medication).

The purpose of this work was to review the effectiveness of pharmacological treatments for apathy in AD. Efficacy, adverse effects, and specific indications of drugs investigated in clinical trials were analyzed comparatively.

## 2. Methods

A systematic literature search was conducted on the PubMed database. Analysis included papers (clinical trials) published from 2003 to 2013 using the following entries: “apathy in AD” “pharmacological treatment of apathy in AD,” “drugs for apathy in AD,” and “medication for the treatment of apathy in AD.” For the entry “apathy in AD” 57 papers were identified, for the entry “pharmacological treatment of apathy in AD,” 24 studies were found, for the entry “drugs for apathy in AD” 4 papers were identified, and for the entry “medications for the treatment of apathy in AD”, 3 studies were found. A total of 88 papers were identified and 14 of them were selected for further evaluation as they fulfilled the “good quality” criteria detailed below. Criteria for inclusion of an article in our review were as follows: (a) the study population should involve patients with AD; (b) the study objective has to evaluate the effects of a pharmacological treatment; (c) apathy represents the primary or secondary outcome of the investigation; (d) the article should be in English; (e) abstract is present; (f) the term “apathy” should be present in the title/abstract (g). Randomized controlled clinical trials (RCTs) were included, whereas review articles were excluded. For nonrandomised studies, the Newcastle-Ottawa Scale criteria that enable to assess quality studies with their design were used [27].

## 3. Results

Treatment of apathy in AD has been evaluated as a primary outcome in 8 studies and as secondary outcome in 6 trials. Collectively the present review has considered 4 RCTs, 9 open-label studies, and 1 retrospective analysis (Tables 1, 2, and 3). Apathy was often assessed as a part of neuropsychiatric symptoms using items such as the Neuropsychiatric Inventory (NPI), the Clinical Global Impression of Change (CGIC) Scale, the Clinical Scale of Neuropsychiatric Symptoms (TOPS), and the Frontal Systems Behavior Scale (FrSBe). A specific questionnaire for apathy, such as the Apathy Evaluation Scale (AES), was used only rarely.

### 3.1. Randomized Controlled Clinical Trials

Of the 4 studies retrieved, 2 assessed apathy as a primary outcome and 2 as a secondary outcome. Among the cholinesterase inhibitors only one study has evaluated the efficacy of donepezil taking apathy as a secondary outcome in patients with AD at an early stage [13]. This work included 153 patients, 96 of whom were treated for 24 weeks with donepezil (5 mg/day in the first 6 weeks and 10 mg/day thereafter). In the active treatment group a higher score at the scale of the apathy compared to placebo was reported. Differences among the two groups investigated were not significant [13]. In this study, treatment with donepezil improved memory tasks, attention, and cognition. Serious adverse events occurred in a similar number of donepezil- and placebo-treated patients [13] (Table 1).

The monoaminergic psychostimulant methylphenidate was effective in apathy in an RCT made in 2008 [22]. The study showed that 13 AD subjects treated with methylphenidate for 2 weeks (5 and 10 mg thereafter) had a greater improvement at the AES total change scores compared with 12 AD patients receiving placebo (end of treatment baseline; Wilcoxon *Z* = −2.00, *P* = 0.045). The NPI apathy scale too revealed a significant difference in scores between methylphenidate and placebo but with less effect (end of treatment baseline; Wilcoxon, *P* = 0.082). Response to methylphenidate was also associated with increase in inattention to a continuous performance task after dextroamphetamine challenge [22]. Adverse events reported were delusions, agitation, irritability, and insomnia [22] (Table 2).

Effects of treatment with *Ginkgo biloba* on apathy were investigated in 400 AD, AD with cerebrovascular disease, and vascular dementia patients treated for 22 weeks with the EGb 761 extract of *Ginkgo biloba* [25]. Subjects treated with EGb 761 showed a significant improvement (*P* < 0.001) at the mean composite score of NPI frequency × severity (21.3 to 14.7 versus 21.6 to 24.1) and the mean score of the NPI caregiver distress (13.5 to 8.7 versus 13.4 to 13.9) [25]. The largest drug-placebo differences in favor of EGb 761 were found for apathy/indifference (statistical significance not reported) (Table 3).

One study has examined the efficacy of the monoaminergic agent modafinil (200 mg/day) on apathetic symptomatology in 23 AD patients treated with cholinesterase inhibitors (Frakey, 2012) [23]. After 8 weeks of treatment, both the experimental (cholinesterase inhibitor + modafinil) and control (cholinesterase inhibitor + placebo) groups showed reductions in apathy on the Frontal Systems Behavior Scale between baseline and final assessments (*F* = 18.017, *P* < 0.001, *η* ^2^ = 0.474) [23]. No significant additional reduction in apathy was found with modafinil (*F* = 0.008, *P* = 0.932, *η* ^2^ = 0.000). The addition of modafinil to the standard treatment for AD (cholinesterase inhibitor medication) did not result in significant additional reductions in apathy [23] (Table 2).

### 3.2. Retrospective Analysis

A retrospective study published in 2007 has investigated the efficacy of citalopram versus the antipsychotic drugs risperidone, olanzapine, quetiapine, and placebo in analyzing the database of CATIE (Clinical Antipsychotic Trials of Intervention Effectiveness in AD) trial [26]. Using data obtained with NPI score, apathy and irritability were investigated in 34 nondepressed AD subjects of the CATIE study. Patients were treated with placebo for 14 days and then with citalopram 5–30 mg for a minimum of 2 weeks and a maximum of 12 weeks (Table 3). Citalopram improved apathy and lowered irritability in the 60% of subjects, but the results were not statistically significant [26] (Table 3).

### 3.3. Open-Label Studies

The effectiveness of donepezil was demonstrated by 3 open-label studies performed including in total 186 patients treated for a period of 12–24 weeks. Apathy was evaluated by NPI or TOPS as a primary outcome measure [14–16] (Table 1).

A study on the prediction of psychiatric response to donepezil (5–10 mg/day) in patients with mild-to-moderate AD has shown that after 12 weeks of treatment the 30% of patients (21 of the 70 enrolled) showed a statistically significant improvement of behavioral disorders (primarily dysphoria, anxiety, and apathy) [14]. The 60% of patients did not respond to the treatment and the remaining 7% had a score statistically worse, particularly in the domain of apathy [14]. The three groups examined did not differ significantly at the baseline in age, education, MMSE, ADAS-cog (Alzheimer's Disease Assessment Scale-cognitive subscale), and Geriatric Depression Scale scores. Statistical parametric mapping analysis of single-photon emission computed tomography (SPECT) images at baseline showed that cerebral blood flow in the premotor and parietotemporal cortices was significantly higher in the responder group than in the worse group [14] (Table 1).

A multicentre study [15] has assessed specific symptomatic changes, including cognitive function, domestic activities, and neuropsychiatric disorders, through a symptom checklist of TOPS. Analysis was completed independently by caregivers and clinicians. The TOPS checklist consists of 19 items and includes a subitem for the evaluation of the overall impression on the symptom apathy. The study has compared neuropsychiatric and behavioral changes for 24 weeks in 101 AD patients treated with donepezil 5–10 mg/day. Apathy was related to changes in standardized scales of cognition (ADAS-cog), activities of daily living (ADL), behavior (NPI), and caregiver burden (CAS). Caregiver evaluation reported that symptoms most improved were cognitive activation, attention, and apathy. Clinician assessments reported that attention was the symptom most often benefiting from treatment followed by apathy. The results at the TOPS checklist correlated significantly with those to standardized measures of cognitive function (ADAS-cog > 50% of the symptoms) (Table 1).

A study of effectiveness and safety in Hispanic patients [16] has evaluated the efficacy of donepezil (5–10 mg/day) in 106 AD patients for 12 weeks. The mean NPI total score was statistically improved from baseline at 6 weeks (−3.2 points, *P* = 0.0018) but not at 12 weeks. The NPI subdomain for apathy/indifference showed a statistically significant improvement from baseline at 12 weeks of treatment (*P* = 0.001) (Table 1).

Two studies with galantamine [17, 18] have evaluated 364 patients for a period of 8–24 weeks. The assessment of apathy was carried out by NPI or CIBIC-plus [17] (Table 1). An observational study, the objective of which was to collect descriptive data on the treatment with galantamine 24 mg/day under naturalistic conditions, has investigated the apathy domain [17]. Most subjects showed no deterioration in behavioral assessments over 3 and 6 months. At 6 months, the majority of patients either improved (range 7.3–28.0%) or remained stable (range 58.7–84.0%). The study did not show statistically significant changes for the evaluation of apathy with behavioral questionnaire CIBIC-plus [17] (Table 1).

Metabolic patterns associated with neuropsychiatric clinical response were investigated in a sample of 19 AD patients [18] treated for weeks with 24 mg/day galantamine. Six patients were classified as responders (NPI score improvement of at least 4 points), 7 did not change, and 6 were classified as nonresponders. After treatment, a significant increase of metabolism was found in the right thalamus of responders. Compared with their baseline values responders showed after treatment with galantamine an increase of the left caudate-thalamofrontal circuit metabolism. In both responders and nonresponders changes in the right ventral putamen were significantly correlated (*r* = 0.63; *P* < 0.05) with improvement in apathy [18] (Table 1).

The activity of rivastigmine was investigated in three open-label studies [19–21] examining on the whole 6,752 patients treated for 24–48 weeks. Apathy was evaluated by Clinical Global Impression of Change (CGI-C) or the NPI score. In the first of these trials effects of rivastigmine were assessed in 173 AD patients resident in a nursing home [21]. Efficacy of drug treatment was evaluated using the NPI-Nursing Home (NPI-NH) test. At 26 weeks, an improvement of the scores for 8 of the 12 neuropsychiatric and behavioral disturbances in patients with specific apathy symptomatology at the baseline was noticeable [21]. The improvement from baseline of the apathy/indifference subitem of NPI-NH was statistically significant (*n* = 37*P* < 0.001). In the second of these trials [20], 2,119 AD patients were treated with rivastigmine (dose at the discretion of the prescribing physicians, 6, 9, or 12 mg/day) for 6 months (Table 1). The results obtained reported an improvement of apathy in approximately 62.6% of patients treated, whereas attention, anxiety, and agitation ameliorated in 67.5%, 62.3%, and 56.0% of patients, respectively (Table 1). In an another more recent and larger multicenter study, apathy was evaluated in 4,460 AD patients treated with rivastigmine 6–9–12 mg/day for 48 weeks [19]. Effects of treatment were assessed by the CGI-C taken as the primary outcome measure [19]. The authors' conclusion was that for each symptom investigated, the percentage of patients experiencing an improvement was greater than the percentage of patients with worsening of symptomatology. Apathy evaluation in eligible patients resulted in the proportions of deterioration improvement/worsening 42.8 versus 7.2% at 24 weeks and 44.1 versus 9.2% at 48 weeks [19] (Table 1).

The effectiveness of methylphenidate was demonstrated by a recent open-label study examining 23 patients [24]. The study has shown a significant improvement of apathy, assessed as primary outcome with the AES (*P* < 0.001) and NPI (statistical significance not reported). Adverse events such as increased blood pressure, hacking cough, dizziness, restless legs, sores in the mouth, arthritic pain in knuckles, and behavioral disturbances consisting of irritability, insomnia, and appetite change were reported. Some of these adverse events disappeared after decreasing methylphenidate from 10 mg twice a day to 5 mg twice a day.

## 4. Discussion

Apathy accompanies a broad range of central nervous system disorders, but its epidemiology, pathogenesis, and treatment options are not clear and often controversial [5–8, 28]. This review on therapeutic strategies against apathy in AD showed that four categories of drugs were used for treating apathy. These include cholinesterase inhibitors, monoaminergic agents (methylphenidate and modafinil), and other different pharmacological profile drugs such as the *Ginkgo biloba* extract Egb 761 and the selective serotonin reuptake inhibitor (SSRI) citalopram. Among treatments compared, modafinil was ineffective and citalopram induced improvements that did not achieve a statistical significance.

Cholinesterase inhibitors were evaluated by 9 studies (1 RCT and 8 open label). These trials did enroll on the whole 7,655 patients, 6,752 of whom received rivastigmine. The global number of patients treated with cholinesterase inhibitors is certainly high, but not the time of evaluation that did not exceed the 6 months. The only RCT study with donepezil showed some nonstatistically significant improvement in the active treatment group. Among the 8 open-label studies with this class of compounds, only 2 reached levels of statistical significance achieved with a *P* < 0.001 in patients treated with rivastigmine [21] and a *P* = 0.001 in donepezil-treated AD patients [14]. On a rather large sample of patients treated with cholinesterase inhibitors (*n* = 7, 655) treatment induced a statistically significant improvement on apathy scores in only 379 (4.95%) patients.

Methylphenidate has been evaluated in 2 studies: 1 RCT and 1 open-label study, enrolling only 36 patients, evaluated for 7 weeks. Effects of pharmacological treatment were significant in the two studies under discussion (RCT study, *P* = 0.045 [22] and open-label study, *P* < 0.001 [24]). Apart from the limited size of the sample, it should be pointed out that the best effects were seen with the dose of 10 mg twice a day that was decreased due to serious adverse events elicited by treatment [22, 24]. The *Ginkgo biloba* extract Egb 761 was evaluated in 1 RCT study on 400 AD patients treated for 22 weeks. It induced a statistically significant improvement (*P* < 0.001) [25] and was well tolerated with adverse events less than those observed in patients treated with placebo in the same trial [25].

Comparing clinical studies reviewed here, the largest sample in which pharmacological treatment induced a statistically significant effect was that of EGb761 with 400 patients investigated, a number too limited considering the large population of AD suffering from apathy. Besides the quantitatively limited evidence of an activity of compounds tested, it should be pointed out that the majority of available studies have several limitations. First, apathy was diagnosed through the reliable AES scale only in 3 studies. This is a relevant issue, as the different components of apathy (emotional, cognitive, and autoactivation) can be addressed only by a specific tool such as AES that is different from the NPI and guarantees a correct differential diagnosis. Second, only a few studies have examined patients with enough characterization and in which cognitive decline was measured and indicated. Third, only a few studies have evaluated the presence of depression, which could have a symptomatic overlap with apathy and may be improved by appropriate treatment with antidepressants. Fourth, only one study has evaluated the neuropsychological functions connected with apathy and in particular executive functions to which apathy is attributed [14]. Executive functions should improve parallel to apathy improvement [14]. Fifth, measurement of effectiveness of pharmacological treatment was done using too many heterogeneous tools such as NPI, AES, and CIBIC-plus and other less common test batteries. This limits the possibility of a comparison among different studies.

## 5. Conclusions

At present apathy accompanying AD is apparently a disorder not largely investigated and its treatment still remains a challenge for pharmacotherapy. Only few treatments were proposed for apathy, but so far there is no clear demonstration of the advantage of one treatment versus others. Therapeutically, several small studies suggested a potential efficacy of various classes of compounds such as cholinesterase inhibitors, monoaminergic drugs, and other compounds with a heterogeneous pharmacological profile. The largest number of subjects was treated with cholinesterase inhibitors that at present may represent a possible option. *Ginkgo biloba* too, on the basis of published results, may be considered as a valid option. For other compounds the small size of samples and some concerns on safety make their role in relieving apathy of no practical importance.

Future research should be carried out using more rigid criteria compared to those used. The main needs are the use of randomized controlled trials and of the current diagnostic guidelines. New efficacy studies should include the evaluation of neuropsychological tests measuring the executive functions which are related to apathy and should also evaluate well-characterized patients, in which the disease severity and the cognitive measures are assessed and reported. Apathy, being one of the most frequent symptoms in AD, needs to be further investigated and its treatment represents a priority.

## Figures and Tables

**Table 1:** Characteristics and outcome of clinical trials using cholinesterase inhibitors on apathy in Alzheimer's disease.

Study	Study design	Sample size	Intervention	Time	Apathy as a primary or secondary objective	Apathy assessment instrument	Outcome
Seltzer et al., 2004 [13]	Randomized placebo-controlled	153 patients	Donepezil (5–10 mg/day)	24 weeks	Secondary	Apathy scale	No difference between donepezil and placebo

Tanaka et al., 2004 [14]	Prospective, open-label	70 patients	Donepezil (5–10 mg/day)	12 weeks	Secondary	Neuropsychiatric Inventory (NPI)	Improvement in apathy in 30% of patients (*P* < 0.01), but significant worsening in 10% of patients

Rockwood et al., 2007 [15]	Multicenter, open-label	110 patients	Donepezil (5–10 mg/day)	24 weeks	Primary	Top Symptoms checklist (TOPS) NPI	TOPS checklist and NPI apathy scores improved

Lopez et al., 2008 [16]	Multicenter, open-label	106 patients	Donepezil (5–10 mg/day)	12 weeks	Primary	NPI	The NPI subdomain “apathy/indifference” significantly improved (*P* = 0.0010)

Brodaty et al., 2006 [17]	Prospective, open-label, observational	345 patients	Galantamine (6–24 mg/day)	24 weeks	Secondary	Clinician's Interview-Based Impression of Change plus Caregiver Input (CIBIC-plus)	Behavior assessment scale stability or improvement of apathy in 87% of patients

Mega et al., 2005 [18]	Prospective, open-label	19 patients	Galantamine (24 mg/day)	8 weeks	Primary	NPI	Galantamine is effective for apathy: change of right ventral putamen metabolic is correlated with change with improvement in apathy (*r* = 0.63, *P* < 0.05)

Gauthier et al., 2010 [19]	Naturalistic, prospective, open-label, postmarketing, observational	3,800 patients	Rivastigmine (6–12 mg/day)	48 week	Primary	Clinical Global Impression of Change Scale (CGIS)	Improvement in CGIS apathy scores; 44.1% (48 weeks) versus 9.2% (baseline)

Gauthier et al., 2007 [20]	Observational	2,119 patients	Rivastigmine (6–12 mg/day)	24 weeks	Primary	CGIS	Improvement in CGIS apathy scores in 62.6% of patients

Cummings et al., 2005 [21]	Open-label	173 patients	Rivastigmine (6–12 mg/day)	26 weeks	Secondary	NPI-Nursing Home (NH)	Decrease in NPI-NH apathy score by 60% (*P* < 0.001)

**Table 2:** Characteristics and outcome of clinical trials using monoaminergic compounds on apathy in Alzheimer's disease.

Study	Study design	Sample size	Intervention	Time	Apathy as a primary or secondary objective	Apathy assessment instrument	Outcome
Herrmann et al., 2008 [22]	Randomized, double blind, placebo-controlled crossover	13	Methylphenidate (5–10 mg/day)	2 weeks	Primary	Apathy Evaluation Scale (AES)	Greater reduction in AES score with methylphenidate than placebo (*P* = 0.045)

Frakey et al., 2012 [23]	Randomized, placebo-controlled	23	Modafinil (200 mg/day)	8 weeks	Primary	Frontal Systems Behavior Scale (FrSBe)	No significant improvement in apathy

Padala et al., 2010 [24]	Open-label	23	Methylphenidate (10 mg/day)	12 weeks	Secondary	AES	No significant improvement in apathy

**Table 3:** Characteristics and outcome of clinical trials using different classes of compounds on apathy in Alzheimer's disease.

Study	Study design	Sample size	Intervention	Time	Apathy as a primary or secondary objective	Apathy instrument assessment	Outcome
Scripnikov et al., 2007 [25]	Randomized, double-blind, placebo-controlled	400	*Ginkgo biloba* extract EGb 761 (240 mg/day)	22 weeks	Secondary	Neuropsychiatric Inventory (NPI)	Significant reduction in NPI apathy scores with *Ginkgo biloba *

Siddique et al., 2009 [26]	Retrospective analysis	421	Citalopram (5–30 mg/day)	12 weeks	Primary	NPI	Use of citalopram was associated with reduced irritability and apathy without sedation
